# Ecological immunology of mosquito–malaria interactions

**DOI:** 10.1016/j.pt.2008.02.008

**Published:** 2008-05

**Authors:** Frédéric Tripet, Fred Aboagye-Antwi, Hilary Hurd

**Affiliations:** Centre for Applied Entomology and Parasitology, School of Life Sciences, Huxley Building, Keele University, Newcastle, Staffordshire, UK ST5 5BG

## Abstract

More than a century after the discovery of the complex life cycle of its causative agent, malaria remains a major health problem. Understanding mosquito–malaria interactions could lead to breakthroughs in malaria control. Novel strategies, such as the design of transgenic mosquitoes refractory to *Plasmodium*, or design of human vaccines emulating mosquito resistance to the parasite, require extensive knowledge of processes involved in immune responses and of microevolutionary mechanisms that create and maintain variation in immune responses in wild vector populations. The recent realization of how intimately and specifically mosquitoes and *Plasmodium* co-evolve in Nature is driving vector molecular biologists and evolutionary ecologists to move closer to the natural setting under the common umbrella of ‘Ecological immunology’.

## Natural vector–parasite interactions in context

A key to interrupting human malaria transmission lies in unravelling the physiological and molecular mechanisms characterizing *Plasmodium*-infected mosquitoes. In the past decade, research on the mosquito immune response has been particularly dynamic, focusing on identifying mosquito genes giving resistance to *Plasmodium* infection using gene mapping, and also focusing on characterizing immune defence mechanisms through gene expression analyses. This area of research benefited tremendously from the completion of the *Anopheles gambiae* genome and from advances in genomics and transcriptomics, such as the development of microarray technology and RNAi gene silencing, resulting in a rapidly expanding body of literature.

Research has also focused on evaluating the fitness costs incurred by mosquito hosts as a direct consequence of infection by *Plasmodium* parasites and the costs and benefits of mounting an immune response to such an infection. From an evolutionary point of view, strong immune defence responses might not always be advantageous to malaria vectors and understanding why some mosquitoes develop an infection whereas others do not could be as important to our understanding of malaria transmission as describing the immune defence mechanisms themselves (Box 1).

For some time the fields of mosquito immunology and evolutionary ecology have relied on laboratory vector–parasite model systems often only remotely similar to natural *Anopheles–Plasmodium* associations but easier to experiment with. However, current research is leaning strongly towards natural vector–parasite systems. There is good reason for this ’return to the field’. For example, more than two decades of research on infection-induced fitness costs using laboratory models have yielded precious few convincing reports and much contradictory data [1]. Gene expression studies of immune responses have suffered a somewhat similar fate, with different mosquito–*Plasmodium* model systems revealing their unique facets rather than suggesting a unified anti-*Plasmodium* response or defence mechanism [2–4]. Thus, results from both specialties emphasize how specifically and intimately mosquito and *Plasmodium* traits co-evolve and underscore the need to work closer to the natural setting [5–8].

## Ecological immunology

The answer to these concerns lies in ‘ecological immunology’, a field of research that focuses on mechanisms and function of immune responses in their ecological and evolutionary context [9,10]. The term ‘ecological immunology’ was first coined in 1996 [9] to describe a growing body of literature produced by evolutionary ecologists focusing on the impact of parasites on host life-history traits, sexual selection and population dynamics. At the core of ecological immunology is the notion that mounting an immune defence is energetically expensive and that individuals must trade-off energy devoted to immunological functions against energy devoted to other life-history activities, such as growth, reproduction and survival [9].

The ecological immunology of mosquito–parasite interactions is a growing area of research that should help us understand how ecological factors affect interactions between mosquito vectors and the malaria parasite to create and maintain variation in host immune defence mechanisms and *Plasmodium* virulence in natural populations. This discipline is benefiting directly from the continuing improvement of research infrastructures in countries endemic for malaria and from a worldwide increase in the number of facilities dedicated to culturing human malaria and experimentally infecting its natural vectors. Here we review what is currently known about genetic and environmental factors affecting mosquito–*Plasmodium* interactions as inferred mostly from experimental laboratory systems and some field studies. This literature is further discussed in relation to population genetic and theoretical studies to delineate promising avenues for future research.

## Parasite and immunity-mediated fitness costs

By their very nature, parasitic infections affect their hosts in adverse ways, which promote the selection of counter measures. However, there is a growing realization that immune systems are themselves costly and that their evolution and successful deployment depends on how they impact life-history traits, such as fecundity and survival, in infected and non-infected organisms [9,10]. Thus, the adaptive advantage of investing resources in immune defences will be related to the virulence of the infection (i.e. the potential extent of parasite-mediated costs) and the risk of exposure in a given population.

### Parasite-mediated costs

Experimental infections of *Anopheles stephensi* with *Plasmodium falciparum* (a natural mosquito–*Plasmodium* association) have shown that parasite-mediated costs begin to be incurred within hours of infection, when nitric oxide synthase (NOS) transcription is upregulated in the midgut epithelium [11]. The transit of ookinetes through midgut epithelial cells induces apoptosis/necrosis in invaded cells, which are subsequently extruded into the gut lumen and replaced by regenerative cells [12]. This model of midgut repair, although likely to be costly, would prevent the development of perforations that could result in increased susceptibility to bacterial infection as suggested by experimental infections of *An. stephensi* with *Plasmodium berghei*, a non-natural host–parasite combination [13]. Midgut invasion was shown to coincide with the transcriptional upregulation of several aspects of the immune system, the downregulation of vitellogenin production in the fat body and resorption of developing ovarian follicles in the non-natural model system *An. gambiae/Plasmodium yoelii nigeriensis* [14,15]. There is also a suggestion that some free amino acids are depleted following infection [16,17]. Different studies based on natural [18] and non-natural associations [19,20] showed that, at a later stage, the presence of sporozoites in the salivary glands increases feeding persistence and probing behaviour, which can result in increased mortality through increased host contacts [21].

Most of these predicted costs have not been directly related to the fate of *Plasmodium*-infected mosquitoes, but several studies have shown that infection sometimes affects survivorship and fecundity (reviewed in [1,22]). Effects of *Plasmodium* infection on mosquito longevity are more likely to be found in unnatural vector–parasite associations and in studies that follow mortality until sporozoite invasion of salivary glands occurs [1]. Although this suggests that longevity could be favoured over egg production in infected mosquitoes (reviewed in [23]), recent studies have shown that infection also curtails reproduction in several mosquito–*Plasmodium* interactions (reviewed in [22]; see also [24,25]). However, most of these laboratory studies were again based on vector–parasite model systems that do not occur in nature (e.g. [24,25]) and thus some of these findings remain to be validated using natural model systems.

### Immunity-mediated costs

Immune responses against pathogens are broadly divided into two categories that differ in their roles, modes of action and costs. (i) Constitutive immune defences are a first line of defence directed against a broad array of pathogens and are constantly activated. (ii) Inducible defences are specific and are switched on in response to a particular threat [26]. The physiological and genetic pathways involved in both types of immune responses are probably also involved in other functions (see, for example, [27]). As a result, an evolutionary change in immune responses might be associated with changes in other traits with which they are genetically correlated. Importantly, if the correlation is negative an increase in immune defences translates into a decrease in other fitness-related traits resulting in an evolutionary trade-off.

Trade-offs are a major cause of constitutive and inducible immune defence costs and, in their simplest form, arise when energetically costly immune responses compete with resources or with a particular gene product required for other important functions [9]. In practice, trade-offs can be driven by complex mechanisms and describing their functions can be challenging. A good description of such trade-off comes from work on *Drosophila melanogaster* in which selection for increased encapsulation ability against parasitoids resulted in constitutive costs in the form of reduced larval competitive ability and lower survival rate [28]. Resistant flies had twice as many haemocytes as susceptible ones, suggesting that resistance depends on investment in haemocytes [29]. Further work led to the identification of two of the major genes underlying this resistance (Rlb and Rat) that could be driving the trade-off between immunity and competitive feeding ability [30]. In *Aedes aegypti*, selection for early and late pupation resulted in correlated changes in body size and melanization response, revealing trade-offs between those traits [31].

In Anophelines, microarray studies have shown that the invasion of the mosquito midgut and salivary glands by *Plasmodium* parasites induces the transcription of several immune-related molecules [3] (reviewed in [32–34]). The production of these molecules is expected to be energetically costly and to divert resources away from growth and maintenance [1]. Nitric oxide (NO) is one such molecule that is part of a peroxidase induction cascade that initiates apoptosis/necrosis in mosquito midgut cells as the ookinetes move through them [35], a process that has been shown to directly or indirectly kill parasites in *An. stephensi* infected with *P. berghei* [36,37]. In this case, inducible costs are expected because NO synthesis requires arginine, a key component of other metabolic pathways such as egg production and sperm maturation in insects that can only be obtained through the insect diet [38]. Another potential cost of NO induction is the autoimmune response it can induce owing to its high toxicity and wide spectrum of action [38].

Similarly, the phenoloxidase cascade responsible for the melanotic encapsulation of parasites in insects produces phenol intermediates that are cytotoxic to the individual [27]. In *An. gambiae*, artificial stimulation of the phenoloxidase cascade and antimicrobial peptide production results in a reduction in egg production owing to the induction of apoptosis in cells of the follicular epithelium and subsequent resorption of eggs [39,40]. However, the exact mechanisms underlying this trade-off are not well understood.

Most of the advances discussed above were obtained through studies based on laboratory models of malaria infection. Consequently, the upregulation of immune genes that these studies describe might not always accurately mirror the upregulation operating in natural associations [2]. Furthermore, these studies do not take into account potentially important genetic variation in response to infection in natural populations. As an example, the strong melanization response of selected *An. gambiae* refractory strains has no match in nature. By contrast, melanization of *P. falciparum* is rarely observed in East African mosquito populations [41] and is observed moderately frequently in West African populations [8].

## Effect of genetic and environmental factors on mosquito fitness and infection

Our understanding of mosquito immunity and fitness costs comes mainly from laboratory studies, but the situation in the field, where environmental stresses will be operating against a background of varied host and parasite genotypes, is probably very different. Here, the outcome of an infection will depend on the mosquito and *Plasmodium* genotypes, on the environment and, importantly, on the way that their genotype responds to changes in the environment; a phenomenon known as phenotypic plasticity (Box 2). For example, even minor changes in environmental temperature can lead to different responses among individuals and, hence, greatly alter the effect of infection [42].

Certain environmental factors (Box 2, Table 1) might influence the outcome of a mosquito feeding on an infective bloodmeal either directly (Box 3), or indirectly, as a result of genotype-by-environment interactions (Box 4). Some of these might alter the expression and success of the immune response directed against the parasite, others will affect the distribution of resources within the mosquito and eventually influence its survival during the parasite developmental period. Although studies of the effect of different environmental qualities on mosquito–*Plasmodium* interactions are not common, *Plasmodium*-induced mortality varies with the temperature [43], diet [44] and density at which adult mosquitoes are kept [45]. However, in a meta-analytic study of several *Anopheles*–*Plasmodium* natural and unnatural interactions, diet and humidity seemed to have an effect on survival in infected mosquitoes wherease temperature did not [1]. In *An. stephensi* infected with *P. berghei*, concomitant bacterial infection [13] also affected *Plasmodium* infections. Finally, in adults of a refractory *An. gambiae* strain, competition at the larval stage strongly influenced melanization response [46] (Box 3). The ability to melanize beads was also affected by adult nutrition [47] and age, with all adults melanizing beads immediately after eclosion but only 23% by the time they were 7 days old [48].

The environment might also influence the expression of resistance if the phenotype is determined by genotype-by-environment interactions, that is if different genotypes respond differently to variation of environmental conditions [42,49] (Box 4). Such an interaction was found in *An. stephensi* mosquitoes infected with *Plasmodium chabaudi*, a non-natural association in which the effect of sugar-water deprivation on infection strongly depended on the genotype of the parasite [44]. However, in another study differences in the concentration of sugar solutions (2%, 4% and 6%) fed to eight isofemale lines of *An. stephensi* infected with *Plasmodium yoelii yoelii* affected infection intensity but did not interact with mosquito genotype [49].

Laboratory experiments are usually designed to test the effect of one stressor, but in the field situation mosquitoes will be exposed simultaneously to multiple stresses that could compound their effects and add an extra dimension to the outcome of exposure to infection. This might be particularly true for parasites that are not highly virulent, for which subtle effects can simply be compensated for by the host, for example by enhanced food intake. In such cases, the major effects of parasitism will only appear when the host is under multiple stresses. For example, immune cost was seen in a refractory line of *An. gambiae* infected with *P. yoelii nigeriensis* when simultaneously subjected to the stresses of induced flight, food deprivation and low temperature [50]. Although one study based on the *An. stephensi* and *P. yoelii yoelii* model system suggests environmental stress negatively affects mosquito survival and infection intensity [49], no study has reported on the effects of environmental stress on fitness cost of *P. falciparum* infection in *An. gambiae* s.s. or *An. stephensi* in the laboratory or in field situations.

## Maintenance of resistance and susceptibility in natural populations

The costs associated with constitutive and (perhaps to a lesser extent) inducible immunity are thought to be the main reason for the maintenance of susceptibility in natural populations [51]. Although alleles for strong constitutive defences might be selected for in laboratory experiments, it is unlikely that these could occur at high frequencies in wild populations. Constitutive defences are thought to be rapid-acting and generally less specific than inducible responses [26], therefore theory predicts that such defences should be favoured when pathogens are highly prevalent in populations and have high growth rates [52]. Inducible defences are expected to be more specific and comparatively less costly and therefore could occur at higher frequency in natural populations [10]. However, this might not always be the case. For example, Armitage *et al.* [53] showed that constitutive investment in prophylactic cuticular melanization in *Tenebrio molitor* fed *ad libitum* did not carry fitness costs, but that an induced encapsulation response affected longevity. One of the obvious challenges of ecological immunology is to identify the ecological factors and genetic mechanisms that determine the investment in constitutive and inducible immune defences and generate variation in these defences among individuals in natural populations.

Sinden *et al.* [5] argue that all mosquitoes should maintain defence mechanisms that have sustainable evolutionary costs and hence they should all be refractory to infection to a certain degree. There is good evidence from laboratory studies suggesting that mosquitoes have baseline defence mechanisms that limit parasite development such that only a small fraction of malaria parasites develop to the sporozoite stage [36,54]. Recent advances also suggest that some wild mosquito genotypes might be resistant to most infections [55,56]. Using mosquito families raised from wild caught females and fed on blood from locally infected patients to ensure a study system similar to the natural system, Riehle *et al.* [56] genetically mapped an ‘island of resistance’ responsible for most of the variation in oocyst number. Genes responsible for melanization were also identified, but melanization only affected a fraction of oocysts in infected females and thus could not account for refractoriness. Instead, a gene encoding for an *Anopheles Plasmodium*-responsive leucine-rich repeat 1 protein (APL1) was responsible for this strong inducible defence against *Plasmodium* [56]. Because refractory and susceptible alleles were found in this study, the important question remains as to what factors determine their frequencies in natural populations.

The environment in which wild mosquito populations live is highly variable in time and in space. There is strong evidence from laboratory systems that genetic and environmental factors interact to determine the fitness of infected and non-infected individuals such that different genotypes might have higher fitness in different environmental conditions (Box 4). Environmental conditions vary spatially and temporally and selection regimes differ markedly, particularly in subtropical and tropical climates typically associated with strong seasonality. For example, large areas of Africa are characterized by dry seasons with low mosquito densities and low malaria transmission, during which the majority of mosquitoes will rarely feed on infected blood [57]. Under such conditions, individuals with lower levels of immunity to *Plasmodium* should be fitter than their resistant high malaria season counterpart, creating seasonal cycles in the frequency of resistance alleles. To date few field studies have attempted to correlate variation in environmental characteristics with variation in immune responses [58].

Another source of variation in immune defence that leads to cycles in allelic frequencies is frequency-dependent selection. Laboratory studies support the idea that the outcome of infection depends on the host and *Plasmodium* genotypes (Box 2, Box 4). Strong genetic interactions between different *An. gambiae* families and natural mixed-infection *P. falciparum* isolates suggest that such interactions could have an important role in nature [59]. In this scenario, parasite genotypes that are most able to invade the most common host genotypes increase in frequency until they become so frequent that rare host genotypes become favoured, resulting in a continual negative frequency-dependent selection process. Theory predicts that under this type of selection regime no single resistance allele should reach fixation [60], hence frequency-dependence could have a fundamental role in promoting and maintaining variation in the immune system.

## Concluding remarks

Despite years of research, relatively little is known about microevolutionary processes that create and maintain variation in immune responses in wild mosquito populations. The ecological immunology of mosquito–*Plasmodium* interactions remains in its infancy and this review should serve as a strong incentive for re-examining the effects of genetic and environmental factors on *Plasmodium* infections using controlled laboratory experiments based on natural mosquito–*Plasmodium* associations. Future transcriptomic and metabolomic studies based on similar experimental designs will further bolster our understanding of the underlying mechanisms leading to variation in immune responses in natural populations. The challenge of the field of ecological immunology is to design tractable field studies that incorporate the multiple ecological factors that are essential for determining the course of an infection. The complexity of the infected mosquito phenotype can easily turn experiments into exercises in confounding factors and correlative interpretations. However, despite these difficulties, describing population-level processes occurring between *Plasmodium* and mosquito hosts remains a priority and a key component in our quest to resolve the malaria problem.

## Figures and Tables

**Figure I:**
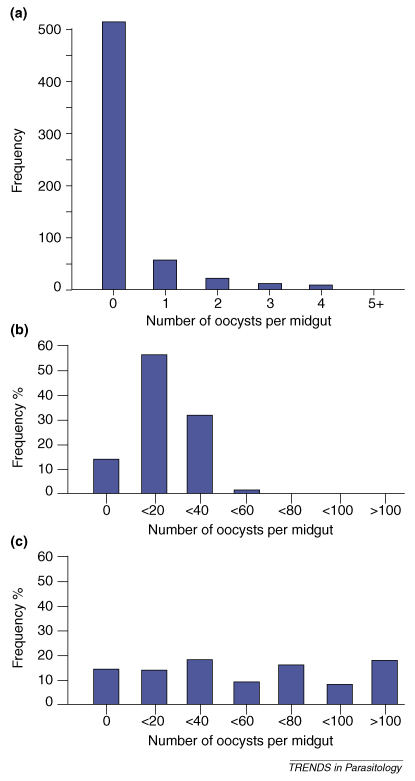
Distribution of *Plasmodium* infections among mosquito hosts. **(a)** Binomial distribution of *P. falciparum* oocysts in a natural population of *An. gambiae* in Northeast Tanzania. **(b)** A normal distribution of oocysts in the ZAN-U strain of *An. gambiae* experimentally infected with *P. yoelii nigeriensis*. **(c)** Uniform distribution of oocysts in the KIL strain of *An. gambiae* infected with *P. yoelii nigeriensis*. The two laboratory strains have been maintained in the laboratory for decades and exhibit high susceptibility to infection. Adapted with permission from [50,61].

**Figure I:**
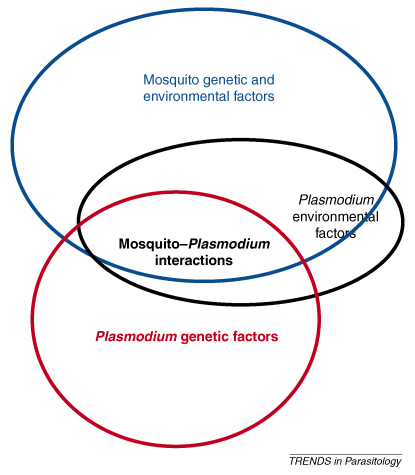
Mosquito–*Plasmodium* interactions as a complex phenotype. Variation in the outcome of an infection is a particularly complex phenotype in that both host and parasite genetic and environmental factors can be considered essential components of each other's ‘environment’ and can strongly influence infection (intersection between blue, red and black ellipses). As an example, the environmental factors affecting *Plasmodium* development inside mosquitoes (black ellipse) are almost entirely determined by mosquito genetic and environmental determinants (blue ellipse).

**Figure I:**
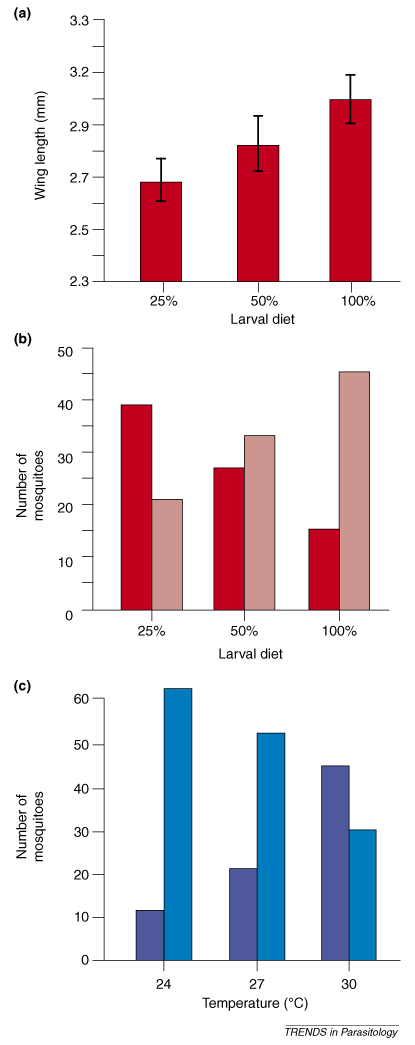
Environmental effects on host body condition and the immune system. **(a)** Phenotypic changes in body size of adult *An. gambiae* females in response to the amounts of food available to the larvae (represented by the percentage of powder food provided). Adult body size is strongly negatively affected by decreasing amounts of resources available at the larval stage. **(b)** Larval food deprivation also affects the intensity of an immune response of adult mosquitoes, as evaluated by the amount of melanization of sephadex beads inoculated in the thorax. Light red bars indicate the number of females with bead melanization ≥50%, dark red bars females with melanization <50%. This is probably because starved larvae give rise to small imagines that are in poor body condition. **(c)** Temperature is another environmental factor that has a strong effect on the immune response to sephadex beads. Light blue bars are number of females with bead melanization ≥50%, dark blue bars females with melanization <50%. Adapted with permission from [46].

**Figure I:**
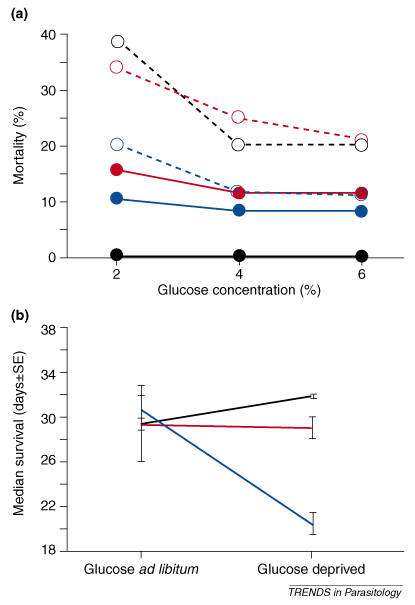
Interaction between genotypes and the environment. **(a)** Effect of food availability (glucose solution) on the survival of different lines (black, red and blue) of *An. stephensi* infected with *P. yoelii yoelii* (dashed lines) or uninfected (solid lines) showing a strong effect of genetic factors on survivorship. **(b)** Changes in the survival of *An. stephensi* infected with different strains of *P. chabaudi* and provided with high and low amounts of food (glucose solution). Infection with two different strains (blue and black lines) results in contrasted norms of reaction to glucose deprivation. Interestingly and predictably, a combined infection with the two strains (red line) gives a response intermediate to the two individual ones. Adapted with permission from [44,49].

**Table 1:** Genetic and environmental factors directly or indirectly affecting mosquito–*Plasmodium* infections

Factors	Possible mechanisms	Expected effect	Refs^a^
**Mosquito genetic factors**			
Genetic determinants of susceptibility and resistance	Immune surveillance molecules (PRRs) immune effector molecules	Parasite survival and development	[34]
Genetic determinants of adult body quality	Adult size and longevity	Parasite development	

**Mosquito environmental factors**			
Larval environment	Temperature, habitat quality, food, larval density	Adult quality, adult longevity, adult immune system, resistance to malaria	[46,63]
Adult environment during infection	Dietary components, temperature, humidity, adult density	Mosquito immune response, survival, parasite development	[1,43,45,47,64]

**Interaction between genetic and environmental factors**			
Interactions with larval environment	Food, temperature, habitat quality, larval density	Adult quality, resistance to malaria	[1]
Interactions with adult environment during infection	Sugar feeding, temperature, humidity	Resistance to malaria and vector fitness	[49,50]

***Plasmodium*****genetic factors**			
Genetic determinants of susceptibility and resistance	Virulence genes, ligands for surveillance molecules (PAMPs), ability to suppress host immune response	Vector survival and fecundity and mosquito immune response	[44,65,66]

***Plasmodium*****environmental factors**			
Mosquito genetic and environmental factors	See mosquito environmental factors within this table and associated references	Parasite survival and development	[1,43,45–47,63,64]
Environmental factors independent of the vector	Temperature and parasite density	Parasite survival and development	[67]

**Interaction between genetic and environmental factors**			
Interactions between *Plasmodium*, mosquito and environmental factors	*Plasmodium* virulence and sugar feeding	Parasite survival and development	[44]

a Where possible, references have been selected to support the concept that genetic and environmental factors will affect infection. Abbreviations: PAMPs, pathogen-associated molecular pattern; PPRs, host pattern recognition receptor.

## Box 1 Distribution of infections among mosquito hosts

As is true for many parasites, in natural populations *Plasmodium* infections are usually binomially distributed among mosquito hosts. Figure Ia shows the distribution of oocysts in a large dataset of wild *Anopheles gambiae* females collected during the rainy season, during high malaria transmission in Northeast Tanzania [61]. Most female mosquitoes were either uninfected or carried few parasites, whereas a few females carried more than five parasites. Although there is no single and simple explanation for this distribution, infective gametocytes in bloodmeals could already be aggregated [62]. Thereafter, heterogeneities in the mosquito and *Plasmodium* population linked to variation in genetic and environmental factors shape the course of *Plasmodium* infections in individual mosquitoes and could account for *Plasmodium* aggregation (Box 2). Individual mosquitoes might also differ in the number of infected bloodmeals they ingest and could acquire one or multiple parasite genotypes, thus further complicating the picture. Mosquito–*Plasmodium* model systems used in laboratory settings feature few of the factors determining patterns of infections in wild populations. Figure Ib and c show contrasted distributions of *P. yoelii nigeriensis* oocysts in experimentally infected females of the ZAN-U and KIL *An. gambiae* strains, two strains that have been maintained in the laboratory for decades and exhibit high susceptibility to infection [50]. For some time model systems involving non-natural mosquito–*Plasmodium* associations were the only option for the experimental study of mosquito–*Plasmodium* interactions. The lack of genetic and environmental variation of experimental systems could explain the uniform distribution of infection observed in the KIL strain. Although model systems facilitated some experiments, they offer limited insights on the processes that create and maintain variation in mosquito immune responses in natural populations, hence current research favours studies conducted closer to the field giving more realistic experimental systems.

## Box 2 Determinants of mosquito–Plasmodium infections

Mosquito–*Plasmodium* interactions are as simple or complex as the fundamental genetic mechanisms underlying them. In simple quantitative genetics terms, phenotypic variation in infected mosquitoes (e.g. survival, immune response level and body condition) can be described as:

(Equation 1) $$V_{P}\;Mosquito=V_{G}+V_{E}+V_{G*E}$$

And similarly, the phenotypic variance of the infecting *Plasmodium* parasites (survival, multiplication, rate of development, etc.) can be summarized as:

(Equation 2) $$V_{P}\;\mathrm{Plasmodium}=V_{G}+V_{E}+V_{G*E}$$

Where *V*_*P*_ is the phenotypic variance among individuals, *V*_*G*_ the genetic variance, *V*_*E*_ the variance owing to the direct effect of the environment and *V*_G*E_ the genotype-by-environment interaction, which occurs when the effect of the environment differs among genotypes. Thus, the phenotypic variance observed among hosts and parasites in natural populations is due to genetic and environmental factors and the interactions between the two. Variation in the outcome of an infection is thus a particularly complex phenotype in that it is determined by factors from both host and parasite equations (Figure I, Table 1). Both host and parasite strongly influence infection and their respective phenotypic variances can be considered an essential component of each other's ‘environment’ that strongly affect their *V*_*E*_ and *V*_G*E_ terms.

## Box 3 Mosquito environmental factors

Factors such as food availability, temperature and humidity or rainfall affect mosquito development at the larval stage and their body condition at the adult stage. Environmental factors largely determine the condition of mosquito hosts when they are infected and probably have a crucial role in determining the outcome of *Plasmodium* infections in nature. Evidence from laboratory models suggests a strong link between environmental factors and the mosquito immune system. For example, Suwanchaichinda and Paskewitz [46] showed that the amount of resources available for larval development affects adult body size and condition, using a selected line of *An. gambiae* refractory to the rodent malaria *P. berghei*. This phenotypic response subsequently negatively affected melanization of foreign particles, the main determinant of infection in the mosquito strain selected for strong constitutive immune response (Figure Ia,b). Ambient temperature also had a strong effect on mosquito melanization response (Figure Ic) [46]. Thus, in natural populations environmental variability probably strongly affects interactions between mosquitoes and their parasites through its influence on host body condition and immune system function. Note that changes in environmental conditions generally affect all mosquitoes, but that particular genotypes could respond more or less strongly to varying environmental conditions. Such interactions are essential for maintaining genetic variation in mosquito immune responses in wild populations (Box 4).

## Box 4 Genetic factors and interactions with the environment

Whether an infected mosquito clears an infection, survives with a given parasite load or succumbs to infection depends largely on mosquito and *Plasmodium* genetic factors, as well as interactions between genetic factors and the environment. Figure Ia shows the negative effects of glucose deprivation in three *Anopheles stephensi* lines either non-infected or infected with the rodent malaria parasite *P. yoelii*. In this particular study there was a strong effect of mosquito genotype on likelihood to survive, but no significant interaction between mosquito genotype and the environment [49]. Figure Ib shows strikingly different effects of two lines of the rodent malaria *P. chabaudi* on the survival of *An. stephensi* females in response to glucose deprivation [44]. Such contrasting mosquito survival rates suggest that the virulence of different parasite genotypes could vary greatly in response to environmental factors. Generally speaking, genetic-by-environmental interactions indicate that different genotypes have higher fitness under different sets of environmental conditions and thus provide a simple explanation for the creation and maintenance of genetic variation in mosquito and *Plasmodium* populations.
